# Males’ Lived Experience with Self-Perceived Pornography Addiction: A Qualitative Study of Problematic Porn Use

**DOI:** 10.3390/ijerph20021497

**Published:** 2023-01-13

**Authors:** Sophia Hanseder, Jaya A. R. Dantas

**Affiliations:** Curtin School of Population Health, Curtin University, Perth 6102, Australia

**Keywords:** pornography, compulsive sexual behavior, qualitative research, heterosexual men

## Abstract

The positive impact of pornography use has been demonstrated; however, most research points towards problematic, compulsive, or excessive engagement with pornography and associated adverse effects on well-being. However, results remain inconclusive and qualitative research capturing perspectives of affected people is scarce. This phenomenological study aimed to explore the perspective and lived experience of males with a self-reported addiction to pornography. Semi-structured in-depth interviews with 13 males aged between 21 and 66 years from Australia and the USA were conducted. A thematic analysis of the transcripts was undertaken, resulting in the identification of four themes. The interviews explored the participants’ reasoning for determining themselves as porn addicts, investigated patterns of use, examined the perceived multifaceted impacts of pornography use, illustrated applied individual strategies to overcome the addiction, and proposed interventions helping to inform future recommendations. Experiences and perceptions of pornography addiction were consistently depicted as problematic and harmful. Most participants described an inability to stop their consumption despite experiencing adverse effects. Commonly reported was a gradual increase in the use of and consumption of new or more shocking content. Consumption of content was outlined as an escape or coping mechanism for negative emotions or boredom. Participants reported a variety of applied strategies to manage their addiction and suggested recommendations. Investigation into strategies for the identification of problematic pornography use, its conceptualization, associated health outcomes, and effective preventative and interventional strategies are required to provide academic consistency, support those negatively affected by pornography, and achieve increased public awareness of the issue.

## 1. Introduction

“Chains of habit are too light to be felt until they are too heavy to be broken.”—Warren Buffet

### 1.1. Pornography Use as a Global Everyday Activity

The increase in online pornography consumption throughout the world, particularly among young males, is enormous and results from modern technological possibilities [1,2,3,4]. Today’s technological advancements make pornographic material globally available and easily, inexpensively, quickly, and discreetly accessible [3,5]. The Internet and smartphones have contributed to the addictive potential of online pornography, considering the “triple-A” impact of accessibility, affordability, and (relative) anonymity, and have thus transformed humans’ understanding of sex and intimacy [1,2,5,6].

The global popularity of online pornography use is represented, for instance, by one pornography site ranking 18th among the world’s most popular 300 websites (while including another 10 pornography sites at other ranks), thus outweighing web pages such as Netflix or eBay [7]. Pornhub, one of the most popular online pornography websites, provides a seemingly infinite choice of pornographic material and counted 42 billion visits in 2019, which equals an average of 115 million daily visits, and this number is rising [5,8]. The USA is the leading nation in terms of its pornography consumption worldwide with a proportion of 30% and 70% of female and male users, respectively, and an average age of users of 39 years [8]. A 2017 Australian study revealed that, without exception, all males within this sample between 15 and 29 years of age had viewed online pornographic material [9]. Of these, 84% of stated that they watch pornography at least once per week, reflecting pornography consumption as a prevailing activity [9,10]. Importantly, most men start to engage in using pornography in childhood or early adolescence [11]. Research indicated a significantly lower number of female users [12,13]. Accordingly, this study exclusively focused on males.

### 1.2. Applied Terminology and Focus within This Study

Definitions of pornography within research are largely inconsistent [14]. Pornography use within this study is understood as the intentional consumption of sexually explicit material, i.e., textual, visual, or audio material, for sexual arousal, autoerotic stimulation, and sexual gratification by depicting sexual activities and/or genitals in unconcealed ways [15,16,17]. As the increase in pornography consumption is believed to mainly result from technological developments, especially the use of smartphones, this study focuses on the predominant use of pornography, i.e., sexually explicit internet movies [4,13,14,16]. Sexually explicit internet movies illustrate the streaming or downloading of pornographic videos over the internet, depicting sexual activities, including a variety of sexual acts and behaviors ranging from activities alone, with another person, or in groups (e.g., masturbation, penetrative and non-penetrative sex, kinks, and fetishes) [4,13,16,17].

The growing popularity of online pornography led to increased attention to the issue within academic and popular literature, accompanied by questions and concerns about its potential harms and adverse effects, particularly for minors and with excessive use [5,9]. For most people, pornography consumption is not believed to be problematic and should hence not be pathologized, as its use may solely be recreational, for exploring one’s sexuality or sexual fantasies, and is associated with hardly any or no attributed negative effects [18,19,20,21]. Instead, several positive effects of pornography consumption have been demonstrated, such as increased sexual satisfaction, confidence, and open-mindedness towards sex and sexuality [9]. However, due to the addictive potential of pornography, there are a significant number of individuals at risk whose use may evolve into a problem and affect their lives, including their physical and mental health, relationships, and social lives, negatively [2,18,19,21]. Accordingly, the present study exclusively spotlighted problematic pornography consumption, acknowledging individuals who perceive their use as addictive, compulsive, or excessive as well as worrisome, harmful, or distressing [5].

Ongoing controversies and, hence, inconsistencies concerning the conceptualization, labeling, diagnosis, and assessment of problematic porn use within the eclectic body of literature resulted in ambiguous research results [21,22]. Accordingly, in line with other key journals, the researcher (the first author—referred to as researcher in the article) applied the terminology of “self-perceived pornography addiction” [2,5,23].

The following definition of self-perceived addiction to pornography was applied for this study and shared with the research subjects: a use of online pornographic material that is perceived as addictive, dysregulated, or compulsive, with associated positive and/or negative effects on one’s life and well-being. Accordingly, the focus of this study was the subjective experience and identification of addictive use of pornography and attributed effects, as opposed to objective measures or a clinical diagnosis [19,24]. Importantly, to avoid bias, the applied definition addressed both negative and positive effects.

Similarly, the term “excessive” was applied as the subjective interpretation of problematic porn use, hence representing a qualitative term rather than an objective characterization [19]. Finally, the researcher declares the application of the outlined terms in consideration of their popularity in the current body of scientific literature, not for indicating any notions towards a particular conceptualization or diagnostic model.

### 1.3. Scientific Controversy about Pornography Addiction

Despite the inclusion of compulsive sexual behavior disorder in the ICD-11 and the category of behavioral addictions in the DSM-5, including hypersexual disorder, the conceptualization, labeling, and clinical recognition of problematic pornography consumption remains inconsistent and controversial [10,21,22,25,26]. In 2019, the diagnosis of “compulsive sexual behavior disorder” was formally added to the ICD-11, consisting of ongoing failed attempts to control one’s sexual impulses or urges and leading to repeated sexual behavior for an extended period [10,26]. Behavioral addiction is usually outlined by a model of problematic use, characterized by aspects such as functional impairment, craving, loss of control, and risky use [2]. Studies reveal conflicting results and researchers are divided concerning the issue [5,27]. Some argued that it had not been proven that pornography use is harmful per se, others highlighted the potential positive outcomes of pornography use, such as sexual satisfaction or stress relief [11,28,29].

The ongoing controversy about the most accurate conceptualization of this phenomenon—as compulsive disorder or addiction—remains, due to a lack of scientific research and inconsistencies of applied definitions and classifications [10,21,27,30,31]. Particularly, the measurement of what is considered problematic pornography use has turned out to be challenging [5,23]. Due to the resulting lack of clinical recognition of pornography addiction and clinical guidelines, there is currently only minimal evidence-based support or treatment options, or knowledge about corresponding efficacy [21].

### 1.4. Risks of Excessive Pornography Consumption

Pornography use is often overlooked or trivialized despite substantial evidence demonstrating numerous potential risks and adverse effects [23,32,33]. This study focused on compulsive, addictive, or dysregulated use of pornography. The results of representative studies that affirm the risks of numerous adverse effects of pornography consumption are profound and must, therefore, be taken seriously [7,15,23]. Importantly, studies outlining the potentially harmful impacts of pornography usually capture excessive pornography use and the addictive nature of online pornographic material, which led to a concerning kind of use [2,23]. Accordingly, certainly not everybody who is accessing pornography is addicted to it. Recognizing the growing evidence that affirms and acknowledges the potential of various harmful effects of pornography consumption is considered crucial, particularly with early exposure [7,23,32,34].

### 1.5. Statement of Significance

The above-mentioned prevalence of pornography use and increasing reports of adverse effects combined with its addictive potential and nearly unlimited availability and accessibility suggest a public health approach. This framework seems necessary in order to develop appropriate policies and interventions [2,7,9,21,35]. Pornography has the potential of affecting fundamental elements of society, including human (sexual) attitudes, behaviors, interactions, relationships, people’s well-being, and health outcomes [36]. Importantly, the COVID-19 pandemic may grant the issue additional urgency, as implemented public health measures, including lockdowns and social distancing practices, were found to have increased global porn use by over 11% [37,38].

Moreover, the growing number of people seeking help through online forums and support communities, or via counselors and therapists, represents a growing demand; hence the need for this study [15,23,39,40]. A lack of clinical recognition can be problematic due to its association with significant emotional distress for people negatively affected by pornography and a lack of treatment guidelines, which may exacerbate people’s psychological well-being [2,23,39].

In summary, the current lack of scientific research and, thus, the shortage of clinical recognition may lead to an insufficient understanding of the issue. The potential risks of increased pornography consumption require great attention, comprehensive assessment, and a call for action to protect children from early exposure, to provide adequate support to people negatively affected by pornography, and to increase knowledge and awareness in the general population, enabling informed decision making. The high prevalence of regular pornography users, an increasing number of people seeking help for self-perceived pornography addiction, and reported multidimensional adverse effects demonstrate the importance of this study. This study may contribute to filling an essential research gap by exploring and identifying related factors, investigating the perceived impact of pornography use, and name counter strategies and public interventions by giving sufferers a voice.

### 1.6. Aim

The main aim of this exploratory qualitative pilot study was to investigate the lived experience of a cross-section of males with a self-reported pornography addiction to enable a comprehensive understanding of the positive and/or negative effects of pornography addiction that can facilitate the development and implementation of preventative and interventional strategies.

### 1.7. Objectives

1. To investigate patterns of pornography use, underlying motivations, feelings, and triggers among self-perceived online pornography addicts.
2. To explore, identify, and assess the multidimensional impacts (emotional, cognitive, psychological, social, sexual, physical, and financial) of pornography consumption in line with self-perceived pornography addiction.
3. To propose strategies as suggested by interviewees to overcome pornography addiction.
4. To develop recommendations that inform future preventative or interventional strategies.

## 2. Materials and Methods

This study applied a qualitative research methodology, guided by a phenomenological approach, exploring the lived experience of male adult self-perceived pornography addicts. Phenomenology attempts to investigate the experiences of a small number of participants that represent transcendental subjectivity, thus enabling a comprehensive understanding of a particular phenomenon through others’ experiences [41,42,43,44]. According to phenomenological research, every interpretation is recognized as a “real” phenomenon and can only be understood with consideration of the individual’s social environment [41,45]. Importantly, the method facilitated the exploration of this phenomenon by providing valuable in-depth insights into the participants’ individual experiences and behaviors, resulting in an increased understanding of self-perceived pornography addiction [41,43,45].

Given the overall aim of this study, enabling an in-depth and holistic understanding of lived experience of males with a self-perceived and self-identified pornography addiction, a phenomenological approach was the most suitable method, as it recognizes subjective perspectives as legitimate and valid [41,45,46]. This was particularly relevant, as pornography addiction is currently not explicitly clinically recognized [10,23,28].

In addition, the semi-structured interviews conducted were based on the four-dimension criteria adapted from Lincoln and Guba [47] (credibility, dependability, confirmability, and transferability) to ensure the quality of this study [48]. According to the knowledge of the researcher, these criteria have never been used in the context of self-perceived pornography addiction before.

### 2.1. Participants

Because of the scientific controversy around the labeling of problematic pornography use as (behavioral) addiction [10,21,23,28], this study exclusively included participants who reported a self-perceived addiction to online pornography (without a clinical diagnosis). Due to the highly subjective nature of self-perceived pornography addiction, screening was performed via self-assessment of participants. Subsequently, participants were asked to state the reasons why they perceived themselves as being addicted to pornography.

Importantly, this study focused on cisgender male participants over 18 years of age, with proficient English, from either Australia or the USA. The study was advertised through posts on respective online forums, namely NoFap and Reboot Nation. Drawing on previous research conducted by the researcher, the selected online forums and communities are well-known and growing in popularity among people who experience their pornography use as problematic. 

Interviews were conducted via voice or video call using the conference platform WebEx. The promotional post contained the aim, objectives, and purpose of the study, conditions of participation, and information regarding confidentiality, anonymity, and ethical considerations. The implication of this recruitment strategy generated a specific group of volunteers that self-identified with an addiction to pornography that is considered problematic, with readiness and willingness to share their experiences with the researcher.

As a small gesture of appreciation and compensation for participation, interviewees received an eBay/Amazon gift card, each valued at AUD $25 (or the equivalent in USD). The gift card was sent to the participants immediately after conducting the interviews. Additionally, any personal details used for the interview process have been destroyed immediately afterward. However, five participants refused to accept a gift card.

### 2.2. Ethical Considerations

Several ethical considerations were assessed for conducting this study safely and purposefully. Due to the topic’s sensitivity and the association with psychological distress [28] and sexual behaviors [33], the researchers ensured a study procedure according to rigorous ethical standards. Ethical approval to conduct the study was granted by the Curtin University Human Research Ethics Committee (HRE2021-0511). Importantly, ethical rigor was assured throughout the entire research process. Liamputtong’s [46] principle of non-maleficence was applied for guidance.

Numerous measures were taken in the study to meet best practice for rigorous ethical standards. Firstly, the nature of the recruitment for participants for the study indicated one’s intrinsic motivation for voluntary participation insofar as those affected had to actively initiate contact with the researcher and express their interest in participation. Secondly, no identifying information was collected, and participants were offered to be addressed with a pseudonym. Moreover, interviewees were informed about their absolute voluntariness of participation in the study by an information sheet sent to them before the appointment of the interview. This information sheet provided them with detailed information about the aims and purpose of the study. It further addressed information about the confidentiality of the collected data and explained the secure and confidential use and storage of the obtained data and its professional destruction after seven years.

The participant information form also described the preservation of anonymity through the process of de-identification of datasets by using codes. Furthermore, participants were informed about their right to pass questions and withdraw from the research process without any consequences until the approval of their transcripts. The transcripts were sent to them shortly after the interviews were obtained, promoting transparency and providing the opportunity to add, clarify, or comment on any collected information [49].

A preamble included in the interview reminded participants of the applied definition of self-perceived pornography addiction as well as the exclusion of reference to any illegal activities, such as illegal pornographic sexual activity. Respective protocols, including the immediate suspension of the interview, were developed, and shared with the participants in case of any disclosure, despite this given information. The researcher summarized the information provided to the participant, and thus ensured that the participant read and understood what he consented to.

The researcher constantly monitored the participants for any signs of distress. A protocol was put in place if a participant would have appeared distressed, including the offer to pause, defer, or end the interview, asking the interviewee about his state of being, providing verbal support, and referring to available support services. The participants and the information they provided were treated with care and respect, always prioritizing the participants’ well-being [46].

Each participant was informed about the interview procedure. Interviews were conducted via WebEx and the host function was applied, enabling only the researcher to record the interview. This way, the researcher ensured that the participants were unable to record or share the material.

### 2.3. Design and Methods of Data Collection

Participants were recruited as previously described, and data collection via interviews commenced. The study conducted 13 semi-structured, in-depth qualitative interviews with males who self-reported to be addicted to pornography, until saturation was achieved [46,50]. According to Saunders et al. [51], saturation in qualitative research is achieved according to the research question. As this study followed a phenomenological research approach, saturation was defined and achieved through interpretative consistency within reported views and experiences [51]. Since this is an explorative study, its scope is limited, and results might not be generalizable beyond this sample. However, due to the strong accordance between the statements of the study participants as well as other studies, results are transferable and significant.

Following the expression of interest of potential participants, the researcher verified their eligibility and provided them with the participant information, which contained a description of the aim, objectives, and purpose of the study, as well as the estimated direction of the interview questions. Furthermore, it provided information about the conditions and framework of the interview process, including explanations regarding confidentiality, anonymity, data usage, and storage. Lastly, the document offered the opportunity for participants to ask follow-up questions.

After the confirmation of the interviewees’ eligibility and their positive answers, the researcher and participants agreed on the date, time, and mode (voice or video call) for the interview.

The interview questions were developed through intensive literature review and the engagement with case self-reports addressing pornography addiction. Following the aforementioned preamble and repeated assurance of the participants’ consent, the interview commenced with a personal questionnaire containing demographic questions addressing age, relationship status, sexual orientation, educational level, and whether people consider themselves religious or spiritual. The researcher ensured that participants were safe, comfortable, and free to speak [52].

Subsequently, the interview consisted of open-ended focus questions and lasted approximately 20 to 70 min (mean duration of 40 min), strongly dependent on the level of detail in the interviewees’ answers and aspects such as personality and comfortableness when talking about the topic of interest [53].

Open-ended but concise interview questions were formulated in close cooperation with the researcher’s supervisor to minimize distress for the participants, enabling them to report their lived experiences with self-perceived online pornography addiction [46]. Questions addressed social, emotional, psychological, physical, and financial aspects of self-perceived pornography addiction. According to the aim and objectives of this study, the interview questions also examined underlying motivations, triggers, individual coping strategies, and proposed public interventions.

Before the interview, the participants provided informed consent. Consent to electronically record the interview was also sought. The researcher provided the interviewees with a document with support services and resources in case they would experience any kind of distress during or following the interview.

To ensure academic integrity and objectivity, an independent transcriber undertook interview transcriptions. Subsequently, the transcripts were sent to the respective participants enabling them to add, clarify, or comment on them [46,49]. This practice served the purpose of ensuring member-checking for precision and accuracy of the interviewee’s statements, guaranteeing authentic representation and, hence, high-quality data collection [49]. However, none of the participants amended their transcripts.

Additionally, the researcher maintained a reflexive journal to critically reflect on her thoughts and observations throughout the research, guaranteeing objectivity, reliability, validity, and rigor [46,50]. Keeping a reflexive journal was particularly useful to identify personal biases and practice the method of bracketing, i.e., the exclusion of the researcher’s prior assumptions, experiences, and knowledge [41,43,46].

### 2.4. Data Analysis

Thematic analysis was undertaken to assess the collected qualitative data of the conducted interviews. Due to the scientific controversy of the topic clear, definitions of terms such as “self-perceived pornography addiction” were indicated and shared with the research subjects [23].

The process of data analysis followed six steps, according to the thematic analysis as outlined by Braun and Clarke [54]. Firstly, the researcher familiarized herself with the collected data by reading and rereading the transcripts. Subsequently, by using the software NVivo 12, an initial coding process described the content. Steps 3 to 5 consisted of the quest for, review, and identification of several themes and sub-themes identified from the interviews conducted. Due to the focus of this study on the individual experiences of the participants, no themes were added by the researcher. As a result, themes exclusively emerged from the data and information provided by the participants. Finally, the analysis report was produced, only reporting what the participants expressed directly. The obtained data was stored electronically, exclusively accessible to the researcher and their supervisor, as outlined in the data management plan. The reflective journal supported the comprehensive analysis by adding thoughts and reflections throughout the research process and avoiding researcher bias [41,43,46]. The analysis was presented in the form of this extended journal article. Intermediate steps and progress were discussed and assessed continuously with the supervisor.

## 3. Results

Thematic analysis of the interview data collected, identified the participants’ reasons for self-determination as pornography addicts, their patterns of use, numerous impacts of porn use, applied strategies to manage or counter one’s addiction, and suggestions for public interventions. The final emerging themes from the conducted interviews are: (1) self-determination, (2) patterns of use, (3) reported impact, (4) individual coping strategies, and (5) suggested public interventions. Except for the first theme, each of them consists of several sub-themes which are illustrated in Table 1. The proposed public interventions were incorporated into the chapter “Recommendations”, as the participants’ statements informed the formulated interventions.

### 3.1. Profile of the Study Population

At the time of the interview, the 13 research subjects who participated in this study all identified as cisgender male and heterosexual and were between 21 and 66 years old (mean age: 32.23 years); the sample was skewed towards younger participants, particularly the Australian participants of the study population. Seven of them stated the USA as their usual country of residence, the remaining six participants resided in Australia. Seven of the participants indicated that they were single, two were married, two were in a de facto relationship, one was engaged, and one was separated. The highest educational level attained by each of the interviewees was as follows: one general certificate of secondary education, two participants with high school diploma, one person with a trade qualification, one with an advanced diploma, seven participants with either a bachelor’s (five) or master’s degree, and one stated to have a PhD. Two of the interviewees considered themselves as either spiritual or religious, two mentioned being undecided, one stated that he was agnostic. The remaining eight participants did consider themselves as either religious or spiritual. The interviewees’ characteristics are illustrated in Table 2.

### 3.2. Theme 1: Self-Determination

All participants described themselves as addicted to online pornography because of the observed difficulty or inability to reduce, limit, or discontinue their pornography consumption. The interviewees reported continued use of pornography despite wanting to stop, identifying their use as unhealthy, or experiencing negative effects.

*I knew it was bad for me, it was very big negative effect on my life, but I just couldn’t stop it. That’s clearly in my eyes a sign of addictive behavior*.

[P2]

Accordingly, most participants mentioned repeated unsuccessful attempts of limiting one’s porn use as well as a compulsive urge to return to using it.

*I think when you want to stop watching it and then you try again and again and again to stop but you keep sort of going back. You feel motivated most of the time to stop looking at it, but it only takes a lapse in motivation, half an hour, one hour, whatever, and then you go back and then you feel rubbish about it afterwards. So, to me that’s like an addiction, when you want to, kind of want to stop doing something but you keep going back and doing it anyway*.

[P13]

Importantly, participants reported feeling *compelled* [P9] to consume pornography. They described their use as *highly compulsive* [P5] and out of their conscious control. One interviewee mentioned that his *willpower’s never strong enough* [P2] to abstain from pornography, another described feeling driven by an *addictive force* [P5]. Some participants also self-determined their porn use as addictive and problematic based on the frequency of use, resulting in the neglect of other areas, and the feeling of pornography *dominating [one’s] life* [P11].

Some participants depicted porn use as a continuum between habit and addiction and many argued that the regular consumption of pornography may only be problematic for people who are prone to addictions.

*It’s like how some people can you know drink alcohol every so often and they’re fine, but for someone else they become an alcoholic, they develop an addiction and I’m like that with pornography, you know, just once is never enough*.

[P13]

Finally, some of the research subjects disclosed physical urges and cravings; one mentioned feelings of restlessness associated with his porn use.

*It’s like a swelling that I felt within my chest. Like I’d be like oh right well I need to calm it down, I need to stop doing it so much but I’d just kind of like start feeling this swelling and this sort of feeling inside me that just like wouldn’t rest. It was almost like screaming at me and before I’d know it, I’d be using again*.

[P12]

### 3.3. Theme 2: Patterns of Use

This theme contains the reported patterns of use of the research subjects, starting from their first exposure to pornography, the development of their use over time regarding frequency, duration, and content, as well as associated motivations, feelings, and triggers.

#### 3.3.1. First Exposure

Participants reported first being exposed to pornography at young ages; one was as young as 2 or 3 years old when, as he reported, his *dad would have the playboy channel on TV while [the family] ate dinner and would constantly have that on* [P11]. The average age of first exposure to pornographic material in this study population was 11.38 years. Contexts of first exposure were explorative and led by curiosity, *just to see what all the fuss is about* [P4], initiated within peer groups or through peer pressure, e.g., by friends or older siblings showing or sending pornographic material, or coincidence, for example by playing online video games or finding magazines or videos from parents.

*I think it was like 13 or 14. So pretty late, so, compared to others. So, someone sent, someone from my school sent me a link to something and I was like, I opened it, I saw it and I was shocked, and I closed it immediately*.

[P2]

Interviewees reported believing that their early exposure impacted their following relationship to pornography, as it appeared to be *“completely normal and acceptable to do that”* [P11] or even expected within their peer group.

*I remember that my friends were using it and were telling me about it, and I told them that I don’t watch any of that stuff and they were just like how can you not if it’s a normal thing to do*.

[P7]

#### 3.3.2. Frequency and Duration

All participants indicated a varying frequency and duration depending on availability and situation but an overall gradual increase in the frequency of use over time, which then steadied and became compulsive or habitual. Some also indicated an increase in the duration of pornography use. The average frequency and duration reported by the participants ranged from once a week to several times a day and from five minutes to *binge*-watching [P9] or so-called *edging* [P13] up to five hours, in which they reported to *just can’t get enough* [P9].

Most interviewees mentioned that the use of pornography was closely linked to masturbation and the goal to achieve orgasm.

*Um, the thing is like pornography was always associated with masturbation. Like the first time I masturbated, it was to pornography. […] Both of those things were incredibly interlinked. There was never a phase of masturbation without pornography*.

[P7]

Additionally, participants elaborated that their mood and feelings had an impact on their use, indicating to be *less susceptible* [P9] to using pornography when *feeling like happy* [P9], leading to the next sub-theme.

*I do remember is that my very productive times of life are associated with very little use of porn and my very unproductive, angry, or anxiety-filled times are, are like associated with heavy use of porn*.

[P10]

#### 3.3.3. Underlying Motivations, Feelings, and Triggers

The interviewees illustrated that their porn use had developed into an *escape* [P10] or *unhealthy coping mechanism* [P2; P13] for negative emotions, such as feelings of inadequacy, loneliness, anger, anxiety, stress, and depression, as well as tiredness, or boredom.

*If you’re stressed-out pornography is an answer. If you’re bored pornography is a solution. If you’re, like kind of been rejected by someone or you feel lonely, pornography’s the answer. They’re like a million different playgirls which is what makes it so insidious I suppose*.

[P7]

Others emphasized the emotions accompanying their porn consumption: an anticipatory excitement before use and, following the use of pornography, negative emotions such as shame, disappointment, disgust, or feeling *a little bit empty* [P1]. These negative feelings after watching porn were echoed by most of the participants.

*Yes, there’s a term online for that exact thing called post nut clarity, where after you’ve finished using it you look at the horrible things that are on your screen and really question who you are as a person. […] A bit of a disgrace, disgust in yourself*.

[P4]

Several participants highlighted their porn use as a preference due to its convenience and *immediate gratification* [P5].

*I mean like someone had just equated it to junk food […]. It’s like laziness in terms of just like, you feel like, you feel like you’re horny or something and you’d rather just want to watch pornography and deal with those feelings instead of going out you know engaging in actual human relationships*.

[P7]

Social media, TV, and advertisements were reported as an important trigger initiating porn use, while participants expressed the criticism that *everything is so hypersexualized these days* [P7], *it’s kind of setting people up to have issues with it* [P11].

*There is a lot of pornography in society. Um, if you walk through the city and see big, big posters of lingerie stores, of women in sexy lingerie or you open a magazine or you open social media or like, TV, pornography is everywhere, just, maybe some, some forms are less extreme than others*.

[P6]

Some participants also mentioned that their *sex education came from pornography* [P10] and using pornography to *get ideas and feelings about sex* [P1]. However, they later realized that porn rather represented a *sexual miseducation* [P1].

#### 3.3.4. Content

Escalation of content was a consistently described phenomenon. The interviewees outlined a development from *vanilla sort of content* [P5] towards more shocking, extreme, edgy, taboo, or novel pornographic material.

*When I started I would get an erection if I would type the word boobs on Google in its search and I’ll see a topless woman and that would be enough. And that just escalated slowly over time to lesbian sex then to regular sex, then to more hardcore types of material like all, it was very unnatural things. It even escalated like to transexual porn and things like, like deep throating and double penetration, stuff like that*.

[P7]

Participants explained this development through a process of desensitization, saying they *didn’t really want to be into that stuff* [P1] or turning to content they previously *had no interest in or [were] disgusted by* [P9]. However, they stated the need for *more and more extreme stimuli* [P4] to achieve the same level of arousal or gratification, as other content would get *boring* [P5]. Some argued that this *chase* [P11] of more shocking and new content would also *perpetuate* [P8] their viewing behaviors.

*You know when your brain takes more and more because you’re just like stimulating yourself with so much intense material your brain takes more and more hardcore material in order to get off. […] And that was definitely much more the hardcore and, stuff that people would not be comfortable with in real life. […] And I needed stuff like that in order to you know masturbate*.

[P7]

The development of porn use was commonly linked with questioning one’s sexual orientation. Many of them turned to homosexual or transgender pornographic material despite identifying as heterosexual. All of them reported that the questioning of their sexual orientation had stopped with abstaining from pornography.

Interestingly, participants reported the escalation of content by applying a language of dependency, comparing their behavior with drug addictions.

*I guess for drug addicts, I think it’s referred to as chasing the high. […] Um, and so I think that that was really what it was for me, was that I was chasing that high and I was seeing pornography as that high and not partnered sex, and so it caused me to go after that high instead of having sex with my partner*.

[P11]

### 3.4. Theme 3: Reported Impact

This theme summarizes the interviewees’ reported impacts associated with their porn use. It contains the following sub-themes: mental health and well-being; sexual attitudes and behaviors; physical health; sexuality and relationships; financial impact; and positive effects.

#### 3.4.1. Mental Health and Well-Being

All participants reported experiencing several adverse outcomes on their mental health and psychological well-being attributed to their porn use. Interviewees stated that pornography would fuel negative emotions and outlined symptoms of anxiety, psychological distress, and depression.

*With the porn use, you could only get aroused with these extreme shocking things and everything other in life seems so dull and it’s just boring. You don’t really enjoy anything*.

[P2]

They also mentioned struggling with insecurity and low confidence and self-worth. One participant disclosed body issues attributed to their porn consumption. Furthermore, expressions about feelings of shame and guilt associated with pornography use were noted. Guilt was commonly related to the impeded productivity experienced, neglect of other areas in life, and the inability to stop using pornography despite identifying negative effects on their and others’ well-being, exemplified by a participant stating that they felt like a *“failure”* [P9].

*Because of just how much damage I did to [my wife], how much it damaged me, how much it’s damaged you know, friends and family. It’s just, it’s, it’s done horrible things to so many people that I care about […]*.

[P11]

Shame was attributed to the actual content that was consumed, whereas others expressed shame in awareness of potentially unethical conditions within the industry of pornography such as *criminal activity* [P9], *sex trafficking* [P1], and *abuse towards women and men* [P4].

*Sometimes pornography is, you know, you know it’s exploitative or whatever and you feel bad about kind of contributing to that by being part of the people that are watching it so. So that, that kind of adds to the shame and self-respect, like, lack of self-respect and self-loathing*.

[P13]

Additionally, several interviewees acknowledged the significant amount of time invested in consuming pornography *at the expense of like other areas of life* [P5], and instead of doing *anything that I’m actually interested [in]* [P9] or, as one participant phrased it, *actually important things* [P5].

*I mean there were some goals which I really was tight when I wanted to achieve them, and I failed at them because of pornography use*.

[P10]

Some participants added that their preoccupation with porn does not only include the actual time of viewing pornographic material but also intrusive thoughts and an intensive preoccupation with *not doing anything but thinking about porn* [P10], hence taking over a significant amount of *mental space* [P10].

*I’m just like, my mind is just in this sort of post pornographic haze that I’m not paying attention to the real world and the environment around me and not interacting with people. I’m just waiting until I get my next fix you know, so it was pretty bad*.

[P7]

Several interviewees elaborated on their extensive porn use, saying it dominated their life, and reported watching porn at work, while walking home, or to fall asleep.

*It consumed my everyday life. […] It was the one thing that was constantly on my mind. I would go on lunch at work, and I would look at porn. I would get home and if my wife wasn’t home yet, I was looking at porn. If she was at home yet I was trying to create situations to where she wouldn’t be in the room, and I could go look at porn. It was, I mean it became, it became my life*.

[P11]

Participants discussed how their porn use resulted in sleep deprivation, as they would often consume pornography *late at night* [P5] and for an extended period, hence negatively affecting their mood and ability to perform effectively. Most research subjects also described a decrease in *levels of motivation and energy* [P7] or feeling *lazy and tired* [P7], leading to a rather lethargic and inactive lifestyle.

The significant amount of time participants preoccupied themselves with pornographic material resulted in the neglect of other responsibilities for some interviewees. Importantly, many participants reported decreased engagement in their social life, self-isolating tendencies and, hence, feelings of loneliness. Some participants reported feeling emotionally *disconnected* [P11], *cutting [themselves] off from other people* [P12], and *fueling detachment* [P1] from human relationships.

*I’ve noticed that you know there’s just a lot of younger guys who kind of just withdraw from society like I did, and you know porn is a huge part of that*.

[P1]

The study population concordantly reported that, with the removal or decrease of pornography use, i.e., after *abstaining from these dopamine highs* [P2], they experienced positive effects on their life and well-being, such as *hav[ing] way more energy* [P6], feeling *more in charge of [one’s] life* [P8] or becoming more motivated, social, and engaged in other areas of life. One stated to *feel like a totally, mentally a different person* [P1] after discontinued porn use.

*Mmhmm, er more energy and being able to invest in you know what’s going on around me. Um, more comfortable with my sexuality like I said. Um, more respectful of women in general. Um, more self-confident. […] Um, more confident in being able to form a relationship definitely, and oh definitely less body insecurity because it’s very distant, distant from that now*.

[P1]

#### 3.4.2. Sexual Attitudes and Behaviors

Participants reported porn had impacted their sexual attitudes, preferences, and behaviors at least to some extent. Some disclosed that their ongoing and long-lasting use of pornography led to objectifying women.

*I kind of assumed that all these women around me just really wanted to have sex and with me as well ‘cause of, ‘cause of the sexual miseducation […], I’d like seen in pornography so I kind of wanted to experience that you know ‘cause you see all these um, different women and you know different types and you want to just make a bucket list*.

[P1]

Some reported prioritizing appearance over personality and *neglecting the [emotional] connection aspect* [P10] within sexual relationships.

*Yeah, I started to prefer er a woman’s physique more than who she really was*.

[P1]

A few interviewees described a clear distinction between sexual preferences and behaviors in porn use and partnered sex.

*I would never want to do this in real life, so like they treat the women and these things, so it’s really strange, so it’s really disconnected*.

[P2]

Whereas others reported having tried to enact pornographic preferences and identified ideas and concepts of sex and sexuality adopted from pornography.

*So, […] the first time I got in a physical relationship with a girl I can now see that I was trying to enact what I used to see on screen. […] And I also remember myself being disappointed when I saw her naked and I can see it was because I was constantly comparing her to what I saw, and I was also disappointed with myself because I was trying to compare myself with the performance on screen*.

[P10]

Some participants expressed that they were unable to determine if and in what way porn has impacted their attitudes and preferences due to long-lasting use.

*Again I think I’m just going to have to say probably ‘cause I don’t, I don’t know what, um, I don’t know what my sexual attitudes or preferences would be without porn*.

[P9]

Finally, some participants described a change of preferences after discontinued use of pornographic material.

*I’m now noticing that you know now that I have been abstaining from it and have kind of started to, I guess overcome the withdrawals and everything of it and kind of get out of its shadow, that some of the things that I thought I wanted are no longer things that I’m interested in or, you know sexually attracted to or wanting to engage in, in partnered sex.*

[P11]

#### 3.4.3. Physical Health

Except for one, all participants reported having experienced *porn-induced erectile dysfunction* [P1; P7]. One participant also stated that he had suffered from anorgasmia in partnered sex. Interviewees substantiated a causal connection between their pornography consumption and sexual disorder by emphasizing that erectile dysfunction would only occur when pornography was removed, i.e., in both partnered sex and masturbation without the use of porn. In addition, participants who achieved abstaining from pornography for an extended period reported their erectile function had returned. Some interviewees also reported having regained spontaneous erections, including *morning wood* [P2], and the experience of *a wet dream* [P3] with discontinued use of porn.

*Physically, there is a, there is a kind of a thing where if you, if you get too used to like masturbating to pornography then you just, it just becomes difficult to do it without it, sometimes you have to take a bit of a break or really cut down on masturbation as well just to kind of get back to being able to do that without pornography.*

[P13]

#### 3.4.4. Sexuality and Relationships

Participants disclosed significant adverse effects on their sexuality and romantic and sexual relationships. Several interviewees elaborated on relational conflicts or loss of relationships due to their porn consumption. The disclosed issues mainly persisted due to secrecy and lying, emotional and physical infidelity attributed to one’s use, and the avoidance of partnered sex resulting from *choos[ing] porn over you know, doing it with a partner* [P11].

*And so behind basically every partner that I ever had’s back, I’d be sex chatting with other people and I would be unfaithful. […] And like in real life as well.*

[P12]

Participants also reported detrimental effects of their self-perceived addiction on their partner’s mental health and well-being, *impact[ing] [her] massively* [P6].

*The damage that I’ve done to my wife is just, it is, that’s what really gives me the most guilt and most shame, is just how I could do that to someone that I chose to marry and, had you know vowed to be monogamous with and to be loyal to and to be there for her and I broke every single one of those. […] It, severely impacted [the relationship]. It, like I said, I did not have sex with her, she would have to, we would constantly get in fights about it. […] It completely shattered her feeling, of self-worth, her self-esteem. Um, it completely destroyed her is I guess really the best way I can put it. Is that it just it shattered her reality, it shattered her world, and it left her empty inside.*

[P11]

The research participants described negative effects on their sexuality by pointing out decreased sexual arousal and low desire, decreased interest and *impaired ability to enjoy* [P3] partnered sex; *getting a bit bored* [P5]; or *not able to get the same pleasure* [P10] as with porn use.

*I’ve basically been in two like long-term serious relationships and throughout both of them I was pretty much using porn the whole time and I think that it becomes a thing that I substitute for sex. So, I think that it lowers my sex drive with my partner.*

[P9]

Some also mentioned having developed performance anxiety *because you see these people doing these crazy things, but I was thinking I don’t know how to do that* [P1]. One participant indicated that he had never experienced partnered sex and attributed that to heightened insecurity because of his porn use.

*All of that kind of sexual side of my life has just been this unhealthy relationship with pornography and not actual proper sort of sexual experiences with people.*

[P13]

Several interviewees expressed similar concerns and stated their regret at having developed ideas and concepts of sex and sexuality through porn as opposed to *making [their] own journey […], to be curious on [their] own* [P8].

*So initially I think you don’t know a lot of things and it was very unfortunate for me that I came to know a lot of things about you know human body and sexuality through pornography. […] The fact that you know the explorations which should have happened in real life happened on the screen and hence you know this, this varying of I want to reproduce a performance. This I think made me suffer a lot in the relationships.*

[P10]

Once again, participants reported positive effects of discontinued porn use, stating to *feel more and more sensitivity coming back, more and more confidence and arousal* [P8].

*My sex drive has, has kind of um, ramped up because it’s not being consumed by porn. So, I’m wanting to have sex more with my wife than I was before […]. Now that I’m engaging in partnered sex it’s a lot more emotional and it’s a lot more exciting and the sensation is a lot better and a lot more enjoyable*.

[P11]

#### 3.4.5. Financial Impact

The majority of the research subjects reported no financial impact of their porn use, as they stated that they exclusively consumed pornography that was *freely available* [P13] online pornographic material. Some stated a slight financial impact, disclosing to have paid for phone sex and webcam girls, purchasing sex toys—to enhance the experience of masturbation—as well as pornographic content via OnlyFans and Snapchat. Only one participant indicated a major financial impact of his self-perceived pornography addiction, including the regular visit of sex workers, webcam girls, and phone sex.

#### 3.4.6. Positive Effects

Most interviewees declared no positive impact of their pornography use, particularly long-term. However, some of them pointed out that it *makes you feel better temporarily* [P3], portraying porn use as a source of sex education and an opportunity to explore one’s sexuality and follow sexual interests and preferences. Furthermore, participants highlighted it as a convenient and enjoyable activity, causing *immediate release of happy feelings* [P7], and as something to connect over with male friends. One participant stated that he thought that pornography can be beneficial and, hence, *has a place in human culture* [P8].

### 3.5. Theme 4: Individual Coping Strategies

This theme contains individual coping strategies participants applied to manage or counter their self-perceived pornography addiction. The sub-themes are: reduced, intermitted, or discontinued use of porn; managing the environment; mindfulness and redirection; and learning and talking about the issue.

#### 3.5.1. Reduced, Intermitted, or Discontinued Use

The main strategy among this study population was the reduced, intermitted, or discontinued use of pornography. Twelve of the thirteen participants reported (attempting) to abstain from porn. Several interviewees referred to the term *rebooting [their] brain* [P6] in this context which, as one participant outlined, often involves the abstinence from porn, masturbation, orgasm, and/or sex for a certain amount of time to *rewire your brain* [P11] and *let your brain recover from all those doses of dopamine* [P6], *transitioning into a life without the use of pornography* [P8].

*So, in so far as you discover and see pornography as an issue, and you find that you have difficulty in not using it, then rebooting kind of becomes the method or even a ritual which you put yourself in and you decide to go through and there are various different like levels or intensities. So, you can abstain fully from everything like even sex and orgasm for a certain period. This is what’s called hard mode. It, it is recommended by a lot of the people in the community because they think that it gives your, your mind and your sexual life a kind of rest, a full rest.*

[P8]

Most participants indicated *the ultimate goal* [P13] would be to *just never do it again* [P9], i.e., abstaining from pornography permanently.

*Yeah, abstain, abstinence that’s the only way. There’s no other, there’s no like I don’t know, the equivalent to like nicotine, that’d be nicotine replacement therapy or something like that. You have to abstain and it’s difficult, it’s very, very difficult.*

[P7]

However, some said they believed that complete abstinence *would be unrealistic* [P5], and that breaks of porn use, such as the *NoFap November* [P4], may be a more appropriate approach. Only one interviewee mentioned aiming to achieve a continued but aware and moderate use of pornography.

#### 3.5.2. Managing the Environment

Participants applied several strategies to avoid triggers and urges for watching pornography by managing their environment and creating barriers to accessing porn. Therefore, several interviewees reported having installed blockers on their devices *to block certain websites* [P13], to avoid social media, or only use *[their] phone or [their] laptop in like downstairs, in public parts of the house rather than like alone in [their] room* [P13]. One participant who mentioned living in a shared house, for instance, explained that they had *moved [their] computer to the main room so neither of [them] are looking at it constantly* [P5]. One participant also mentioned switching off his devices to reduce his screen time overall.

#### 3.5.3. Mindfulness and Redirection

The interviewees disclosed *to replace porn with things* [P1], i.e., other desired activities, to redirect triggers and urges, and, hence, finding healthier coping mechanisms for stress, boredom, and negative emotions. Accordingly, participants described practicing mindfulness to develop an awareness of one’s triggers, enabling them to redirect these triggers.

*One of the big things […] is um, trying to find your triggers and trying to find ways to cope with urges. And the one that I found that works best for me is distraction. So, when I have an urge or I get triggered, instead of acting on that urge I will find something more constructive to do and I will choose to do that instead of acting on the urge and engaging with pornography use.*

[P11]

Some interviewees reported *turning [their] life upside down* [P8], *changing [their] whole life* [P2], and *many habits* [P2] aiming for an overall healthier lifestyle by concentrating on enhancing their social life, exercise, diet, and sleep.

*And in general, I do think that like having kind of a holistic, you know improve everything approach is the best. So just like you know working on any other kind of bad habits or things in my life. You know I want to eat better; I want to have a better sleep schedule […].*

[P9]

One participant also mentioned practicing Karezza to return to a satisfactory partnered sex life (additional note: Karezza is a sexual practice that is not focused on orgasm but on loving attention, intimate connection, and an affectionate bonding experience, with reported health and relationship benefits [55]).

*It’s just a great way to connect and be supportive and be loving. It helps with arousal issues because you, you know take away that pressure to perform and you know you don’t have to worry about having an orgasm and making your partner have an orgasm, you can just enjoy loving with each other and it’s great*.

[P3]

#### 3.5.4. Learning and Talking about the Issue

Many participants mentioned the use of different resources to educate themselves about the issue. Several interviewees mentioned having read books or having informed themselves on different websites, online forums, videos, and apps to learn more about the issue and accomplish self-help. Most research subjects also found it helpful to talk about their self-perceived pornography addiction. Some participants reported talking to close people in their lives, such as partners, family members, or friends. Others reported attending self-help meetings or other support groups to *talk to other people struggling* [P12] and *supporting each other and trying to help each other, yeah, with recovery* [P9].

Additionally, the use of online forums or, rather, the collection of forums, i.e., subreddits, namely Reddit, NoFap, Reboot Nation, Your Brain on Porn, and Porn Free, was perceived as useful by the majority of the interviewees. They described their use of these forums to access resources, read, share experiences with others who were negatively affected by pornography, find accountability partners, or keep a journal.

Participants indicated that these online forums helped them by providing a *sense of community* [P7] and *learning about the various theories and experiences people have had* [P8], hence normalizing the issue through meeting people who are *experiencing the same kind of, of emotions and the same kinds of situations that [they] find [themselves] in* [P11]. Most participants perceived the use of online forums as *beneficial* [P13] and facilitative to counter one’s self-perceived addiction.

However, one interviewee criticized these forums by saying that some of the members’ attitudes or advice was dubious. Another participant specifically criticized the website Reddit, illustrating the contradiction of included support groups for self-perceived pornography addicts as well as porn sharing communities on the same platform.

*The problem with that is that it’s basically on a porn site. I mean Reddit has so much porn it’s ridiculous so if you, you know if you download Reddit app for example and you’re a porn addict and you’re trying to just be on your like porn recovery community. I don’t know it would be like trying to have an Alcoholics Anonymous in a bar or something you know*.

[P9]

Finally, a few participants mentioned having sought professional help from a counselor or therapist and finding it *super helpful* [P12].

### 3.6. Recommendations

As this study aimed to engage the study population in the identification of relevant public health interventions, the last theme thus presents proposed recommendations informed by the participants’ statements. It persists of the following sub-themes: education and increased public awareness; regulated access; and increased research expenditures and adequate support.

#### 3.6.1. Education and Increased Public Awareness

All participants concordantly addressed the need for increased education and raised public awareness of the issue. They addressed the demand for developing and implementing education programs for the public as well as within schools, and reported they *weren’t really taught about it at all* [P5] but believe *the information should be made available earlier in […] life* [P4].

*[…] Not just teenage boys probably girls as well, with just raising awareness for people that age that you know this can be an addictive thing and you can end up also seeing things which you don’t feel good about or can be disturbing or whatever and there needs to be much more awareness*.

[P13]

Many participants called for increased education and awareness within society about the potential of addiction and other associated risks of pornography use.

*I think there needs to be more education on its harms. It’s a bit, it’s a bit similar to, ‘cause it’s been compared to the tobacco industry from back in the day where people aren’t aware of what it’s doing to them and so many people are doing it*.

[P1]

Some interviewees expressed that we should also challenge the normalization of online pornography in Western societies due to its potential harms, because *people don’t know how much damage it can do* [P6], with particular focus on the porn industry and potentially unethical and illegal occurrences.

#### 3.6.2. Regulated Access

Many interviewees believed porn is *too easy to obtain currently* [P4] and accordingly expressed the necessity for *some legislative measures* [P1] enabling access control and limited availability.

*I don’t think it should be easily accessible. I think it shouldn’t be the default that it just can come flooding into your house if you click a few things, you know I think that’s ridiculous. I think one of the reasons why nothing changes, why this epidemic of it just goes on is because so many people are using it*.

[P3]

Some participants stated *that porn should be banned* [P2] and not be *legal in any way, not even the slightest er, most innocent form* [P6]. However, others explained to *think it’s not feasible* [P1] to do that. They *would like to see it happen, but […] don’t think it’s worth pursuing* [P1]. However, one interviewee pointed out that not everybody who is consuming pornography becomes addicted to it. Consequently, he stated believing that for the people for whom porn may be *a positive thing in their life* [P10], it should be still available, but people would need to *underst[and] the risks* [P5] and *that excess of anything is bad* [P10].

*Yeah, so I think pornography is like cigarettes. I do not think it should be banned. I do not think it should be, it should be promoted as well but it should come with a label on the pack saying excessive use is injurious*.

[P10]

However, most interviewees expressed the need to implement measures of *age verification* [P1] *to protect children from it* [P3] and prevent early exposure.

#### 3.6.3. Increased Research Expenditures and Adequate Support

Extended academic research expenditures and *public health funding* [P7] are required to clarify current scientific controversies, generate more information, and thus enhance and develop evidence-based support and treatment options.

*Unfortunately, from what I’ve read and what I’ve experienced it seems like there is a, a big divide in, you know in psychology where you have a lot of people that you know are, I guess you know the more sex-positive and they don’t view porn as, as a problem and they see it as just normal thing. But then you have the other side where they do see the damage that it does, and they do see the psychological effects and the long-lasting effects that it has on people and how it can be an addiction and really cause some serious, serious issues*.

[P11]

Interviewees emphasized that *people need to know about this […], so we can be better equipped […] to deal with this* [P11]. Some interviewees stated that, currently, there is *very limited* [P4] support and *it’s quite difficult to find* [P4]. However, participants also acknowledged that there seems to be a change in which people start *to become more aware of the harmful effects now and the fact that this can be an addictive thing* [P13] and *are […] waking up to this and adding to the knowledge base* [P10]. Therefore, more research should be conducted to meet this group’s unmet need for safe and effective support for their self-perceived pornography addiction.

### 3.7. Summary of Findings

This study contained a cross-sectional of 13 cisgender males, of an average age of 32.23 years, from the USA or Australia, with a self-reported pornography addiction. The study population self-determined being addicted to pornography by referring to the inability to reduce or discontinue one’s consumption despite experiencing negative effects of one’s use which, thus, felt like it was out of their conscious control. The participants reported believing that porn use may only be problematic for some people.

The average age of first exposure in this group was 11.38 years and occurred in various contexts. All of them described a gradual increase in frequency and some in the duration of use over time. The average frequency ranged from once a week to multiple times a day; average duration ranged from five minutes to several hours. The interviewees’ porn use commonly developed into an unhealthy escape or coping mechanism and individual sessions were often followed by negative emotions. Most participants described an escalation of content guided by shock and novelty towards more extreme content as a process of desensitization.

Participants elaborated on numerous and multifaceted adverse effects of their porn use affecting their mental and physical health, their sexual attitudes, and behaviors, as well as their sexuality and relationships. For the majority, there were no financial impacts associated with their pornography consumption. Positive effects were acknowledged; however, these were identified as only temporary. All men described significant improvements in their symptoms with abstinence from pornography.

The participants applied several strategies to cope with their self-perceived addiction. The main coping strategy applied was to reduce, intermit, or discontinue one’s porn use. The participants applied strategies to identify and then manage, reduce, and replace triggers and urges, for instance, by more desired activities, frequently making major changes towards a healthier overall lifestyle. Many interviewees also educated themselves about the issue and talked about it with different important people in their life, such as partners, family members, or friends, by using online forums, or seeking professional help.

The participants proposed recommendations focused on increased education and public awareness, regulations for the access of pornography enabling informed decision-making, and age verification to protect minors from early exposure. Finally, increased research expenditures were considered required to generate evidence enabling the development of providing safe and effective support and treatment to people suffering from pornography consumption.

## 4. Discussion

This study explored the lived experience of males with self-reported addiction to pornography. The main findings of the study, as outlined in the summary, include reported symptoms and applied language of dependency, early ages of first exposure (average age: 11.38 years), a gradual increase of the quantity of use, escalation of content, and the development of porn use as a coping or escape mechanism. Furthermore, this study presented reported, multifaceted, and mainly detrimental effects of the participants’ porn use on their lives, individual applied strategies to manage or counter one’s self-perceived addiction, as well as proposed recommendations concerning public interventions. Despite the ongoing controversy about the conceptualization, labeling, and approach of problematic porn use, the results of this study are well-documented and -supported by an existing body of academic literature [2,5,9,21,23,56].

### 4.1. Addiction Framework

Participants reported multifaceted and severe adverse effects attributed to their porn use, impeding their health, social life, relationships, sexuality, and general well-being. All research subjects described their relationship to pornography within an addiction model, referring to a compulsive habit and high-frequency viewing behavior, the inability to control and failed attempts to reduce or stop one’s use, the experience of withdrawal symptoms, cravings, and urges with abstinence, and the continued consumption despite experiencing adverse effects. Importantly, the self-determined evidence they provided for porn being the reason for the reported negative impacts was substantiated by the claim of the improvement of all described symptoms with discontinued porn use and may hence provide relevant implications. Additionally, participants indicated the belief in the concept of “addictive personalities” by identifying themselves as prone to addictions and acknowledging that most people may have no problems using pornography healthily and beneficially [39]. Considering the data collected, conceptualization of problematic porn use within an addiction framework seems useful and appropriate.

### 4.2. The BERSC Model

Nonetheless, results of the literature on the topic remain inconsistent and causal pathways unclear, resulting in an ongoing debate and divide within medical/mental health scientific communities [21,23,57,58]. This ongoing controversy keeps people negatively affected by pornography and practitioners from receiving and, respectively, providing evidence-based assessment, support, and treatment [21,58]. Due to the ongoing debate surrounding the issue and the high levels of shame and distress identified with it, the authors suggests the application of the BERSC (i.e., biological, emotional, relational, social, and cultural) model developed by Hall [58]. Hall [58] proposed a biopsychosocial approach, as opposed to a disease model, to address porn addiction in a non-pathologizing and holistic way, to ease current controversies, and focus on the professional responsibility of providing support to sufferers. Additionally, due to the oft-highlighted moral component of self-perceived addiction, objective measures with little reference to subjective feelings of distress may be insufficient, hence requiring a more comprehensive perspective [1,25].

The BERSC model enables an integrative approach for assessment and treatment considering the client’s psychological experiences, contextual aspects, and cultural sensitivity, while recognizing those elements as equally important [58]. This model may be helpful, particularly as it accounts for the multifaceted nature of reported adverse effects in a bidirectional way [58]. For instance, applied to the relational aspect of the BERSC model, low relationship satisfaction may be the cause or, conversely, the consequence of problematic sexual behaviors [58]. Therefore, the application of this model may contribute to exploring causal pathways of problematic porn use as well as recognizing and supporting the group of people negatively impacted by porn.

### 4.3. Education and Increased Awareness

Notably, academic literature and the participants of this study highlighted the need for increased education and public awareness to acknowledge and understand the issue of interest [23,59]. For the majority, the use of pornography may be unproblematic and have positive impacts [9,11,12]. However, for a group of the population, including the cross-section of participants within this study, the use of pornography may become problematic [2,11,18]. The research subjects reported believing that their early exposure had impacted their later relationship to pornography and expressed regret for not having had the chance to explore their sexuality by themselves “in real life”. All research subjects reported early ages of first exposure (average: 11.38 years). Several studies highlighted a significant association between early exposure and negative impacts of pornography use [12,23].

Most males are known to engage in pornography in childhood or early adolescence [11,59]. Consequently, children, particularly, must be protected, as there is currently hardly any protective mechanisms or regulations to accessing online pornographic material [7,60]. Dawson et al. [59] called for sex education tailored to the needs of children and adolescents of today. Accordingly, they expressed the need for including topics contained in porn in school-internal sex education programs by focusing on relevant sexual health topics such as body image issues, sexual and gender-based violence, or the fetishizing of gay and transgender communities. It is considered crucial to reduce the shame associated with porn use but increase children’s critical thinking skills and challenge messages in pornographic material to enable realistic expectations and perceptions of sex and sexuality and, hence, a healthy and fulfilling sex life [59].

### 4.4. Research Implications

This study confirmed common use patterns and adverse effects found in previous key studies [23,56,61], outlined applied individual coping strategies, and proposed public interventions that may inform future preventative and interventional strategies. The ongoing controversy may lead to inadequate or lacking support and treatment due to the remaining absence of acknowledgment and clear evidence concerning the issue. The experiences of people with self-perceived addiction to pornography should not be minimized or dismissed, as this could further increase feelings of shame and exacerbate their health outcomes [5]. The identified results may provide invaluable insights and implications for future research and enable a comprehensive understanding of the lived experience of males with self-perceived pornography addiction.

### 4.5. Strengths

This study provided timely and qualitative evidence that the addictive, compulsive, or dysregulated consumption of pornography has the potential to cause adverse effects on one’s life and well-being. Awareness about what pornography represents, entails, and might cause is believed to be crucial for enabling people to make a conscious and informed decision regarding their pornography consumption. The findings of this study provided insight into the lived experience of males who perceive their pornography use as addictive. The exploratory qualitative approach facilitated an in-depth exploration and understanding of pornography users’ patterns of use, motivations, feelings, and triggers, numerous effects of their porn consumption, and applied individual interventions, providing rich qualitative data. The participants’ experiences revealed useful implications informing recommendations for the development and implementation of strategies improving (sexual) health outcomes and personal well-being within the community.

Additionally, the rigorous application of the four-dimension quality criteria guaranteed the quality and robustness of this study [48]. The credibility of the results was ensured by prolonged and comprehensive engagement with the research topic before conducting the interviews, to establish and enhance the researchers’ knowledge, and by providing the transcripts to the participants [48]. The researchers demonstrated dependability by providing a detailed description of applied methods as well as an audit trail for ensuring the study’s reproducibility. Furthermore, triangulation was assured, as the researcher developed a scoping review on the same topic, kept a reflexive journal, and engaged in regular debriefing with her supervisor, guaranteeing confirmability. Finally, transferability was guaranteed as saturation was achieved through interpretative consistency.

### 4.6. Limitations

However, this study also has several limitations. The study used a cross-section of male participants, self-reported data, and small sample size. Due to the subjective nature of this study, results may be prone to biases and, hence, have some limitations. There is the risk of researcher bias as both authors identify as female whereas the study population was male. This potential limitation was managed by practicing researcher reflexivity, keeping a reflective journal, regular debriefing with the supervisor, and member checking. Additionally, from the researcher’s experience, prior research on respective online forums, and direct feedback from the participants, most males considered it easier to discuss their pornography use and associated feelings with females than males. The researcher has not found any academic resources concerning the preferred gender when talking about pornography use. However, a study conducted by Liddon et al. [62] showed that males preferred female therapists slightly over male therapists.

Moreover, the topic of this study is considered highly sensitive and is associated with high levels of shame; hence, there is the risk of social desirability bias. The previous extensive engagement with the subject and providing a high level of anonymity sought to ensure a safe, non-judgmental, and comfortable environment. In addition, the study utilized self-reporting and the participation of volunteers, and is thus prone to recall and volunteer bias, which was accounted for when interpreting the results. Importantly, the sample size of this study was small, and participation was restricted to male English-speaking adults from Australia or the USA, and is thus not representative of the population, limiting the generalization of findings. Due to the applied recruitment method, participants’ reports on their self-perceived porn addiction are likely to be shaded by the perceptions, terms, and concepts used on these forums or subreddits. It is also possible that cultural differences were not sufficiently appreciated and considered in the data analysis. Finally, by using online forums addressing pornography addiction for recruitment and relying on the participants’ self-assessment, there is a risk for selection bias and potential benefits of pornography use may be underrepresented. Future studies are necessary to confirm the results of this study and include the participants’ sexual, psychosocial, and medical history as well as cultural considerations.

### 4.7. Suggestions for Future Research

The identification of a gradual progression of porn use to become addictive, compulsive, or dysregulated may possess important implications for the identification of risk factors and warning signs. Additionally, this study focused on adult males older than 18 years of age. However, as early first exposure is believed to be a significant risk factor or indicator for adverse effects associated with porn use, and given the participants’ young ages of first exposure, future research should also focus on the impact of porn use on children and adolescents. Given the participants’ statements concerning their partners’ detrimental effects, it is advisable to also generate insights about their experiences. Future follow-up studies are recommended due to ongoing technological developments and are expected to provide valuable implications.

## 5. Conclusions

This study was pursued to explore the lived experience of adult males with self-perceived pornography addiction. It generated evidence that problematic use of porn is associated with multifaceted detrimental effects on one’s life and well-being and can be an essential step towards the identification of the experiences and needs of the affected. This study provided increased knowledge and understanding of self-perceived pornography addiction by exploring the participants’ reasons to self-identify as addicted, their patterns of use, attributed effects, applied individual coping strategies, as well as proposed public interventions. Further research is imperative, and the development and implementation of effective preventative and interventional strategies is required to protect children from early exposure, provide negatively affected people with adequate support and treatment, and enable adults to engage in informed decision-making about their pornography use. Given the globally high and increasing use of pornography and the growing number of people seeking help concerning a self-perceived pornography addiction, a public health approach to prevention and interventions is appropriate.

## Figures and Tables

**Table 1:** Summary of themes and sub-themes.

Themes	Sub-Themes
Patterns of use	First exposure Frequency and duration Underlying motivations, feelings, and triggers Content
Reported impact	Mental health and well-being Sexual attitudes and behaviors Physical health Sexuality and relationships Financial impacts Positive effects
Individual coping strategies	Reduced, intermitted, or discontinued use Managing the environment Mindfulness and redirection Learning and talking about the issue
Recommendations	Education and increased public awareness Regulated access Increased research expenditures and adequate support

**Table 2:** Participant table—summary of participants’ demographic information (*n* = 13).

Participant	Gender	Age	Relationship Status	Sexual Orientation	Educational Attainment	Usual Country of Residence	Religion
P1	Male	21	Single	Hetero	HSD ^1^	USA	Undecided
P2	Male	27	Single	Hetero	HSD ^1^	Australia	No
P3	Male	66	Married	Hetero	Master’s	USA	Spiritual
P4	Male	25	Single	Hetero	Bachelor’s	Australia	No
P5	Male	26	Single	Hetero	AD ^2^	Australia	No
P6	Male	27	De facto	Hetero	TQ ^3^	Australia	No
P7	Male	27	Single	Hetero	Bachelor’s	USA	No
P8	Male	31	Single	Hetero	Master’s	USA	Yes
P9	Male	30	De facto	Hetero	Bachelor’s	USA	Agnostic
P10	Male	38	Separated	Hetero	PhD	USA	No
P11	Male	42	Married	Hetero	Bachelor’s	USA	No
P12	Male	34	Engaged	Hetero	GCSE ^4^	Australia	Undecided
P13	Male	25	Single	Hetero	Bachelor’s	Australia	No

^1^ High school diploma. ^2^ Advanced diploma. ^3^ Trade qualification. ^4^ General Certificate of Secondary Education.

## Data Availability

The datasets produced and analyzed for this study are not publicly available.
